# Current Perspectives on Viable but Non-Culturable (VBNC) Pathogenic Bacteria

**DOI:** 10.3389/fpubh.2014.00103

**Published:** 2014-07-31

**Authors:** Thandavarayan Ramamurthy, Amit Ghosh, Gururaja P. Pazhani, Sumio Shinoda

**Affiliations:** ^1^National Institute of Cholera and Enteric Diseases (NICED), Kolkata, India; ^2^Collaborative Research Center of Okayama University for Infectious Diseases in India, NICED, Kolkata, India

**Keywords:** bacteria, viability, culture, VBNC, resuscitation

## Abstract

Under stress conditions, many species of bacteria enter into starvation mode of metabolism or a physiologically viable but non-culturable (VBNC) state. Several human pathogenic bacteria have been reported to enter into the VBNC state under these conditions. The pathogenic VBNC bacteria cannot be grown using conventional culture media, although they continue to retain their viability and express their virulence. Though there have been debates on the VBNC concept in the past, several molecular studies have shown that not only can the VBNC state be induced under *in vitro* conditions but also that resuscitation from this state is possible under appropriate conditions. The most notable advance in resuscitating VBNC bacteria is the discovery of resuscitation-promoting factor (Rpf), which is a bacterial cytokines found in both Gram-positive and Gram-negative organisms. VBNC state is a survival strategy adopted by the bacteria, which has important implication in several fields, including environmental monitoring, food technology, and infectious disease management; and hence it is important to investigate the association of bacterial pathogens under VBNC state and the water/foodborne outbreaks. In this review, we describe various aspects of VBNC bacteria, which include their proteomic and genetic profiles under the VBNC state, conditions of resuscitation, methods of detection, antibiotic resistance, and observations on Rpf.

## Introduction

The viable but non-culturable (VBNC) state is a unique survival strategy adopted by many bacteria in response to adverse environmental conditions. When the VBNC concept was proposed some 30 years ago, many issues related to its importance were raised, since there was no demarcation between dying cells and adaptive strategy made by the bacteria to cope with stressful conditions (1). The VBNC state of bacteria was previously referred as a dormancy state. However, several investigations showed that the difference between VBNC and dormant stages is based on their metabolic activity (reviewed in Ref. (1)). In VBNC state, the metabolic activity is measurable, whereas, in the dormant state, this activity shall remain below the detection levels. Extensive molecular studies over the years have resolved most of these issues and now VBNC has been accepted as a distinct survival state of bacteria (2, 3). The physiological consequence of VBNC/metabolically active but non-culturable (ABNC) state could be an adaptive effect that supports long-term survival under unfavorable conditions or an after effect of cellular deterioration, which conserves specific features of viable cells but results in a loss of culturability with available techniques. This state can be thought of as an inactive form of life waiting for revival under suitable conditions. In case of many medically important bacteria, this appears to happen spontaneously either when they are present in environments/foods or in the human body during the infection process. Although what triggers this is unknown, it is established that bacteria in VBNC state can resuscitate under “appropriate” conditions.

Viable but non-culturable pathogenic bacteria are considered as a threat to public health and food safety due to their non-detectability by conventional food and water testing methods. Hence, the identification of VBNC bacteria and understanding their mechanisms of their survival and persistence in the environment are of utmost clinical importance. Several pathogens in this condition may contribute initially to non-apparent infection, which are followed by disease manifestations weeks or months later.

Several human pathogenic bacteria undergo morphological changes (aberrantly shaped cells) when they enter into the environments (4–6). For example, *Vibrio cholerae* transformed into coccoid cells in an aquatic microcosm after incubation for 60 days at 4°C (7). In this form, the bacteria remain in the VBNC state and cannot be cultured by conventional laboratory methods. In the VBNC state, many pathogens cannot be cultured by conventional culture methods and this may sometimes lead to an underestimation of their population density.

## Are VBNC Cells Living and Virulent?

Many effective markers have been proposed to confirm that VBNC cells are alive. These include confirmation of cellular membrane integrity, uptake of labeled amino acids, and protection of genomic DNA (5). For *V*. *cholerae*, membrane integrity and colonizing ability of VBNC cells were confirmed in the gut of iron-dextran-treated mice (8).

It is still not clear whether the infectivity of stressed VBNC cells is only a transient process or an indication of active infection. *Shigella dysenteriae* type 1 is a highly infectious organism responsible for causing acute dysentery in humans. With the existing culture techniques, detection of this pathogen from the environmental samples is difficult due to its low prevalence and/or its rapid entry into the VBNC state. In the VBNC state, *S. dysenteriae* type 1 continue to retain Shiga toxin encoding gene (*stx*) in the functional form and produce biologically active toxin (9). VBNC cells showed adherence to the Henle 407 cell line, but lost their invasive property, which is a hallmark of the infection. It was shown that *Vibrio alginolyticus* and *Vibrio parahaemolyticus* VBNC cells lose virulence after their resuscitation in the mouse, which, however, could be restored after two consecutive passages of the strains in the rat ileal loop (10).

It has been observed that different pathogenic bacteria display several manifestations of their virulence attributes in the VBNC state. In the non-culturable state, *Aeromonas hydrophila* lost its ability to lyse human erythrocytes and adhere McCoy cells. After temperature-induced resuscitation (from 5 to 23°C), however, it regained its virulence properties (11). In another enteric pathogen, enterotoxigenic *Escherichia coli*, heat-labile or heat-stable enterotoxins, were not expressed after a prolonged period of incubation in the seawater (12). When *Listeria monocytogenes* strains remain in the aquatic environment in a VBNC state, they are not pathogenic to human adenocarcinoma cell line HT-29 and in the mouse model (13). Similarly, the VBNC state of *L. monocytogenes* from seafood has not shown any virulence either in the cell plaque assay or when injected intraperitoneally to immunodeficient RAG1 mice (14). The non-culturable cells of *Francisella tularensis* were found to be avirulent in mouse model studies (15).

The VBNC *L. monocytogenes* induced after exposure to distilled water showed virulence after they were resuscitated using embryonated eggs (16). The laboratory induced VBNC cells of *E. coli* O157:H7, which is an important pathogen that causes hemolytic uremic syndrome, produced Shiga-like toxins in the vero-cell microplate cytotoxicity assay, demonstrating that this pathogen from the environmental waters/foods could be a potential health hazard (17). Thus, several aspects need to be examined before deciding whether a pathogen in VBNC state is pathogenic or non-pathogenic based on the type of the assay and variation among strains.

## Proteomics and Transcriptomics of VBNC Cells

When the bacterial cell enters into the VBNC stage, it undergoes many cellular changes, which include cell leakage, depletion of energy pools, altered expression of genes, and DNA replication (18). Other factors such as low nutrient content, low oxygen availability, and impairment of a particular metabolic pathway have also been implicated under this transition stage.

It was seen that, after seven days of incubation, *Helicobacter pylori* cells entered into VBNC state and transformed into coccoid shape. In this stage, *H. pylori* produce more alkaline phosphatase, but less urease, leucine arylamidase, and naphthol-AS-beta-1-phosphohydrolase (19). In closed systems, *E. coli* transited from the culturable to VBNC state due to the release of organic molecules into the medium (20). Further, it was seen that the use of “conditioned” media (supernatants of the culture media in which *E. coli* cells were grown previously), prolonged the duration of the VBNC state. The viability of *E. coli* O157:H7 10 months after its induction into the VBNC state by river or chlorinated waters has been demonstrated by establishing the presence of mRNAs of *rfbE* and *fliC* genes that encode parts of O-antigen transporter and flagellar filament structural proteins, respectively (21).

In *E. coli* (EHEC) O157, resistance to oxidative stress has been generally found in isolates from foods while those of the patients remained susceptible (22). By using the proteomic analyzes, it was shown that the VBNC cells of the food isolate had decreased levels of oxidation-responsive factors (AhpCF and AceF), but exhibited a distinct increase in the outer-membrane protein W (OmpW) levels (23). Ribosomal activity is an important function of all the living cells. In VBNC cells, the expression levels of ribosome-associated proteins such as ribosomal-associated inhibitor (RaiA), 40S ribosomal subunit (S6), and bacterioferritin comigratory protein (Bcp) have been low compared to those in the normal cells.

The VBNC cells of *Vibrio vulnificus* highly expressed glutathione S-transferase as determined by suppression subtractive hybridization of differentially expressed genes (24). Under *in vitro* conditions, the normal viable cells of this pathogen were susceptible to oxidative stress in the presence of H_2_O_2_ and to 1-chloro-2,4-dinitrobenzene. In the VBNC cells, accumulation of this antioxidant-glutathione prevents damage to cellular components caused by the reactive oxygen species such as free radicals and peroxides. Compared to normal *V. parahaemolyticus*, several up-regulated proteins associated with transcription (homologs of alpha subunit DNA-directed RNA polymerase and phosphoribosylaminoimidazole carboxamide formyltransferase/IMP cyclohydrolase), translation (ribosomal protein S1, homologs of elongation factor TU, and elongation factor EF-G), ATP synthase (F1 alpha subunit), gluconeogenesis-related metabolism (dihydrolipoamide acetyltransferase and glyceraldehyde 3-phosphate dehydrogenase), and antioxidants (homologs of peroxiredoxins, AhpC/Tsa family) were identified after stimulation and/or maintenance of the cells under VBNC state (25).

It has been known for many years that there is a strong association between *H. pylori* infection and peptic ulcer/gastric cancer. Since *H. pylori* survives well in biofilms, there exists a possibility of its transmission through drinking water. Like many other bacterial pathogens, *H. pylori* has the ability to enter into the VBNC state in the aquatic environment. The conversion of typical *H. pylori* cells into coccoid form of VBNC state has been shown to be accompanied by changes in OMP patterns (26). In addition, under this condition, alpha-ketoglutarate oxidoreductase (KOR) activity is enhanced while the CoA transferase activity is reduced in the Krebs cycle. Based on these observations, it was proposed that it should be possible to kill *H. pylori* strains resistant to multiple antibiotics by direct inactivation of KOR, thereby preventing the cells from entering the VBNC state (27).

The global transcription pattern of almost all the bacterial groups of VBNC state showed that the modulation was mostly in those genes responsible for the cellular processes (8, 23). The VBNC *Staphylococcus aureus* displayed mutational inactivation of catalase (KatA) or superoxide dismutase (SodA) encoded by the genes *katA* and *sodA*, respectively. These genetic changes rendered the bacterial cells highly susceptible to seawater stress at 4°C, thereby preventing their resuscitability (28). In the VBNC state of *V. cholerae*, expression of several genes that encode DNA polymerase II (*polB*), flagellar motor switch protein G (*fliG*), flagellin subunit protein C (*flaC*), and iron(III) ABC transporter encoding gene (VC0230) was found to increase (8). It has also been reported that VBNC *V. cholerae* expresses *tcp* encoding the toxin co-regulated pilus required for the colonization in the intestines of infant mice (4). This finding suggests that TCP may be one of the important factors for the adaptation and survival of *V. cholerae* during its transit from the host to the aquatic environment and *vice versa*. Further, the VBNC state cells expressed the cholera toxin gene (*ctxA*) confirming their virulence in this state (29). *Campylobacter jejuni* expresses its virulence-associated genes (*flaA, flaB, cadF, ciaB, cdtA, cdtB*, and *cdtC*) poorly in the VBNC state (30).

The ClpX is a hexameric-ring ATPase that binds and denatures substrate proteins such as sigma factor RpoS. Disruption of *rpoS* that encodes the alternative sigma factor RpoS σ(38) caused rapid induction of *Salmonella enterica* serotypes Oranienburg and Typhimurium to the VBNC state (31). In *S. enterica* seorvar Typhimurium, mutation in the *clpX* gene was found to be responsible for delaying the cells from entering into the VBNC state (32). Involvement of the stationary phase sigma factor RpoS in the persistence of VBNC state has been demonstrated in several species of bacteria. Guanosine 3’,5’-bispyrophosphate (ppGpp) acts as a positive regulator during the production and functioning of RpoS (33). Compared to the parent strains, the mutants of RpoS die quickly and cannot be activated by existing resuscitation methods.

Cell wall chemical composition (peptidoglycan) of *Enterococcus faecalis* VBNC cells showed an increased presence of higher oligomers (trimers, tetramers, and pentamers) compared to the normal cells (34). Changes were also seen in penicillin binding proteins (PBPs), enzymes involved in the terminal stages of peptidoglycan assembly, and in the autolytic enzymes, with an increase in the activity of latent muramidase-1. Expression of the outer-membrane protein CadF, which mediates in the binding of *C. jejuni* with extracellular matrix protein fibronectin of Caco-2 cells, has been detected in these cells after their entry into the VBNC state (35). This finding indicates that even in the VBNC state, some pathogens can maintain their virulence irrespective of the modifications in some of the cellular protein structures. In the VBNC state, cell wall of *V. parahaemolyticus* becomes porous allowing cells to empty their cellular space. In this condition, the cells showed tolerance to heat, H_2_O_2_, and low salinity, but were susceptible to bile salts (36).

Alkyl hydroperoxide reductase subunit C (AhpC) is the catalytic subunit responsible for the detoxification of reactive oxygen and facilitates the survival of pathogenic bacteria under environmental stresses or during infection. In *V. parahaemolyticus*, *ahpC1* (VPA1683) and *ahpC2* (VP0580) are located in chromosomes II and I, respectively. These *ahpC* genes were shown experimentally to protect the pathogens against extrinsic peroxides H_2_O_2_ and tert-butyl hydroperoxide (tBuOOH) thereby prolonging the time taken by cells to enter into the VBNC state (37). It was also seen that in this pathogen, expression of VP2468 (*dacB*), which encodes d-alanyl-d-alanine carboxypeptidase, was more during the cold induction of VBNC. The process was accompanied by the disruption of cell wall synthesis resulting in aberrantly shaped cells (38).

In some bacteria, such as *H. pylori* and *V. fluvialis*, the genetic backgrounds of the parent strains remain unchanged even after the cells enter the VBNC sate. This suggests that the genomes of the bacteria do not undergo radical change when they enter the VBNC state, even though the cells undergo morphological and other metabolic changes (19, 39).

## Resuscitation of VBNC Bacteria

### Conditions that induce VBNC state

Several factors such as antibiotic pressure, high/low temperature, starvation, chlorination, change in the pH, and oxygen stress can induce VBNC state of any bacteria (5, 35, 40). The culture supernatant of ameba *Hartmannella vermiformis* was shown to induce the VBNC state of *Legionella pneumophila* (41). This may be due to the nutrient depletion caused by the growth of *H. vermiformis* coupled with products of metabolism present in the medium. The VBNC cells of *C. jejuni* failed to resuscitate in human intestinal epithelial cells *in vitro*, but retained their capability to invade Caco-2 cells (30). The exact mechanism of support rendered by the eukaryotic cell lines to the VBNC bacteria has not been studied in detail.

### Conditions that help in resuscitating from VBNC

Favorable growth conditions with a source of energy and an ideal stoichiometric ratio of carbon to inorganic elements can reverse the VBNC state. This phenomenon is widely known as resuscitation. Several environmental factors are known to minimize the possibility of pathogenic bacteria’s entry into the VBNC state. It was observed that under low salinity conditions, presence of humic acids significantly reduced loss of culturability of several enteric bacteria (42).

Visible light (40 Klux) is a strong inducer of the VBNC state. Gourmelon et al. (43) studied the involvement of oxygen and reactive oxygen species in preventing the phototoxicity of *E. coli*. They found that catalase, which eliminates hydrogen peroxide, thiourea, a hydroxyl radical scavenger and desferrioxamine B, and an iron chelator, were effective in reducing phototoxicity.

Some of the human pathogens, such as *E. coli* O157:H7, can be resuscitated using its autoinducers such as 2(A1-2) produced during biofilm formation in a serum-based medium (21). Combinations of amino acids such as methionine, glutamine, threonine, serine, and asparagine in the basal minimal medium are also known to support the resuscitation of *E. coli* cells (44). Intracellular human pathogen *L. pneumophila* is a bacterial parasite of many species of freshwater protozoa. The VBNC cells of this pathogen were resuscitated in a supportive medium with reactive oxygen scavengers such as sodium pyruvate or glutamate or with cultures of *Amoebae castellanii* (45). Sodium pyruvate restores the biosynthesis of macromolecules such as DNA and proteins, thereby converting the VBNC cells to a culturable state (46).

In *E. coli*, a dual-component system, EnvZ/OmpR plays a major role in mediating signal transduction in response to osmotic stress; EnvZ is a transmembrane histidine kinase that monitors environmental osmolarity and OmpR is a response regulator. Darcan et al. (47) demonstrated that *E. coli* could not enter the VBNC state under the stress of osmolarity, pH and starvation in the absence of the EnvZ product.

The embryonated egg model was found to be useful in resuscitating the pathogens, *L. monocytogenes* and *C. jejuni* from the VBNC state (16, 48). Several enteric pathogens induced artificially to the VBNC state by exposure to cold conditions were resuscitated by co-culturing them with several eukaryotic cell lines (49). Epidemic strains of enteroaggregative *E. coli* serotype O104:H4 that harbors the *stx* have been isolated from the water after the addition of copper-ion chelating agents, which facilitated resuscitation of this pathogen (50). The VBNC cells of *Pseudomonas aeruginosa* could be resuscitated by the addition of the chelator diethyldithiocarbamate and the revived cells exhibited cytotoxicity to the CHO cells (51).

In *S. enterica* serovar Typhi, Zeng et al. (52) found that the addition of Tween 20 (3%) or catalase (1%) allowed the VBNC coccoid cells to become culturable again within 24–48 h. In addition, the resuscitated cells remained virulent as evidenced by the animal model studies. Studies conducted with environmental *Salmonella enteritidis* demonstrated sodium pyruvate as the key molecule in the resuscitation process (46). Under *in vitro* conditions, organic molecules released into the medium due to the growth of a bacterium may play a role in the conversion of culturable to VBNC state (20). However, the metabolic products that act as resuscitative agents have not been studied well. The bacterial protein, YeaZ was found by Aydin et al. (53, 54) to act as a classic actin-like nucleotide-binding protein and resuscitate VBNC *V. parahaemolyticus* and other enteric bacterial pathogens.

## Detection Methods

Lack of proper methods of detecting the pathogens in VBNC state increases the risk of human exposure to contaminated water/foods. Hence, for checking the level of microbial contamination, it is very important to use appropriate techniques for the detection of VBNC bacteria and establishing their viability. Whether the bacteria under VBNC condition are alive or not can be proved by using several criteria, which include cellular membrane integrity, uptake and incorporation of labeled amino acids, protection of the genomic DNA from DNase I digestion, and global gene expression (5, 18). Several methods used in the detection of VBNC bacteria are shown in Table 1. It has been noticed that sometimes VBNC cells, which are unable to grow on agar plates, can recover their ability if plated on matrices other than agar. The reason for this has been attributed to the presence of trace quantities of 5-hydroxymethylfuran-2-carboxylic and furan-2-carboxylic acids in agars (55).

**Table 1:** **Methods for the detection of VBNC bacteria**.

**Method**	**Indicator/function**	**Bacteria**	**Reference**
Acridine orange staining, fluorescent antibody-direct viable count (DFA-DVC)	Elongation of cells in the presence of nalidixic acid or ciprofloxacin	*Vibrio cholerae* O1 and other Gram-negative rods	(56, 57)
*p*-Iodonitrotetrazolium violet assay	Activity based on electron transport system	*Shigella dysenteriae* type1.	(58)
CTC and DAPI double staining	Respiratory activity	*Campylobacter jejuni, Vibrio parahaemolyticus*, and *Listeria monocytogenes*	(10, 59, 13)
Metabolic activity	Accumulation of rhodomine	*Francisella tularensis*	(15)
Growth on matrices	Absence of 5-hydroxymethylfuran-2-carboxylic and furan-2-carboxylic acids	*Pseudomonas collierea*	(55)
Quantitative PCR (qPCR)	Global expression genes	Vibrios, *E. coli*, etc.	(60–64)
qPCR with propidium monoazide	Binding of propidium monoazide with extracellular DNA in dead or membrane- compromised cells inhibits qPCR amplification	*Legionella pneumophila*	(64)
RT-PCR	Quantification of 16S rRNA	*Bifidobacterium* spp.	(65)
DVC-FISH	Measure of RNA, DNA and mRNA changes	*Helicobacter pylori*	(66)
MALDI-TOF/MS	Identification of differentially expressed proteins	*Vibrio harveyi*, *Enterococcus faecalis*	(67)
Solid phase cytometry and fluorescent viability staining	Identification based on intracellular esterases	*C. jejuni*	(68)
RING-FISH	Detection of individual genes	*V. parahaemolyticus*	(69)
Biosensor	Detection of beta-d-glucuronidase	*E. coli*	(70)
SELEX technique	DNA aptamer-based viability detection	*Salmonella enterica* serovar Typhimurium	(71)
Bacteriophages	Lytic activity of live cells	*E. coli, Salmonella* spp	(72, 73)
Eukaryotic cell extracts	Supplementation with TCBS	*V. cholerae* O1	(74)

Initial studies on VBNC bacterial detection relied on modified cell staining procedures. The Kogure procedure is based on acridine orange staining of elongated cells in the presence of DNA-gyrase inhibitors such as nalidixic acid (56). In course of time, however, many bacterial pathogens became resistant to nalidixic acid necessitating modification of Kogure’s method. In many studies, ciprofloxacin has been used in order to accommodate both Gram-negative and Gram-positive bacteria in the screening. Due to the prevalence of high-level resistance to nalidixic acid in *V. cholerae* in cholera-endemic areas, a modified fluorescent antibody-direct viable count (DFA-DVC) technique with ciprofloxacin has been used to detect VBNC state of this pathogen (57).

Double staining with 5-cyano-2,3-ditolyl tetrazolium chloride (CTC) and 4,6- diamino-2 phenylindole (DAPI) has also been used in the detection of VBNC and total bacterial cells. In the DAPI counterstaining, cells fluoresce in blue allowing proper contrast, permitting simultaneous enumeration of both total and viable bacteria using a single filter (13). LIVE/DEAD BacLight bacteria viability kit (Molecular Probes, Eugene, OR, USA) uses nucleic acid stains, green-fluorescent SYTO^®^ 9 and red-fluorescent propidium iodide. These stains differ with their ability to penetrate healthy bacterial cells. SYTO^®^ 9 stains both live and dead bacteria, while propidium iodide penetrates only the bacteria with damaged membranes and reduces the SYTO^®^ 9 fluorescence when both the stains are used. With the mixture of these two stains, bacteria with intact membrane fluoresce green, while dead bacteria with damaged membranes fluoresce red. Because of its discriminatory power, this kit has been used in direct fluorescent microscopy, quantitative assays with a fluorescence microplate reader, and flow cytometer/fluorometer.

Since the bacterial cells in the VBNC state are difficult to culture, PCR with *Vibrio*-specific 16S rRNA-directed primers has been performed using the total DNA extracted from the water samples followed by denaturant gradient gel electrophoresis (DGGE) and qPCR for quantification of *Vibrio* population (60, 63). It is not possible to differentiate dead from live bacteria by normal PCR or qPCR, as these methods target only the DNA. To circumvent the problem, ethidium monoazide has been used to quantify DNA selectively from viable cells, including the VBNC bacteria in normal membrane/cell wall of aquatic environment, using real-time PCR (61). Propidium monoazide is a microbial membrane-impermeable dye that binds to extracellular DNA and DNA in dead or membrane-compromised cells and inhibits qPCR amplification. Considering these properties, propidium monoazide has been used in the detection of many human pathogens (64, 75). VBNC cells maintain high levels of rRNA as the normal cells and retain reductase activity, which are essential for all living cells and because of this, quantification of 16S rRNA has been used in several RT-PCR assays. RNA-based genotypic approaches targeting stress related genes have been made to identify VBNC cells of *Salmonella* spp. in biosolids qPCR (76). For the detection of *V. cholerae* in the VBNC state, 16S rRNA and mRNA for *tuf, rpoS*, and *relA* have been targeted (77).

A combination of modified direct viable count method using novobiocin as the DNA-gyrase inhibitor and fluorescent *in situ* hybridization (FISH) was successfully used for the detection and enumeration of *H. pylori* in the VBNC state in freshwater/seawater samples (78). Using matrix-assisted laser desorption ionization time-of-flight mass spectrometry (MALDI-ToF/MS) along with multivariate data analysis, Kuehl et al. (79) could distinguish between resuscitated and non-culturable cells of *E. faecalis*.

Quantification of bacterial loads is an important parameter in drinking water disinfection processes and supply. Solid phase cytometry in conjunction with fluorescent viability, biosensor with Moraxella-modified glassy carbon electrode, and flow cytometry in combination with different fluorescence dyes are some of the tools used (68, 70, 80). Since the pathogenic strains of *V. cholerae* go into the VBNC state, it will be difficult to isolate them by conventional culture methods. The pathogenic strains of *V. cholerae* O1 were recovered from several water samples after allowing them to grow in thiosulfate citrate bile salts sucrose (TCBS) agar supplemented with eukaryotic cell extracts, which are thermostable and proteinase-K sensitive (74).

Recognition of individual genes by fluorescence *in situ* hybridization (RING-FISH) is a technique that can detect individual genes in a single bacterial cell and thus can, unlike the conventional FISH which targets abundant genes, help enumerate the number of bacteria in a sample even if their number is low. A highly sensitive aptamer-based viability sensing technique has been developed by Labib et al. (71). In this method, DNA aptamers highly specific to the target pathogen have been selected by systematic evolution of ligands by exponential enrichment (SELEX) technique, then the specific sequence, which displayed the highest binding affinity with the live cells was integrated into an impedimetric sensor via self-assembly onto a “gold nanoparticle modified screen-printed carbon electrode.” Several recent studies have shown the usefulness of bacteriophages in the detection of VBNC cells present in the bacterial populations (72, 73).

## Effects of Antimicrobials

The VBNC state of several pathogens such as *E. faecalis, H. pylori*, and *Haemophilus influenza* has been found to be resistant for several antimicrobials (3). Benzylpenicillin, piperacillin, and gentamicin inhibit peptidoglycan or protein synthesis of *E. faecalis* in the VBNC state and impede its resuscitation (81). *S. aureus* presents as biofilms can cause recurrent infection and can enter the VBNC state in the presence of vancomycin or quinupristin/dalfopristin when these drugs are used for treatment (40). During the process of infection and in the course of treatment regimens, certain bacterial cells can transfer into the VBNC state. In this state, the bacteria are generally resistant to multiple antimicrobials and this may cause treatment failure at times (82). A good example of this phenomenon is the drug resistance of tuberculosis. The non-culturable *Mycobacterium tuberculosis* is multi-drug resistance and may prolong the infection and chemotherapy for long time (82).

## Public Health and Environmental Investigations

Several physico-chemical and ecological factors such as light, temperature, high or low salinity, pressure, and low nutrient content can induce the VBNC state. In addition, protozoans and zooplankton are known to support VBNC bacteria in the aquatic environments. In the food industry, processing the food/drinks, improper pasteurization, and pressure of CO_2_ while packing have also been shown to induce a VBNC state in bacteria (83). In clinical settings, pathogens in VBNC state have been found to be responsible for many latent infections, which may show up after months or even years (e.g., tuberculosis).

Biofilm formation is a process that represents the most successful adaptation of bacteria against several environmental factors. It has become increasingly evident that biofilms in drinking water supply systems provide a transient or long-lasting habitat for many microbes, including the human pathogens. Remarkably, part of the biofilm population of pathogenic bacteria persists in a VBNC state and escapes detection by the existing methods. The presence of *Staphylococcus epidermidis* in the VBNC state leads to the formation of biofilms, which are not only tolerant to many antimicrobials, but also contribute to immune evasion (84).

*Legionella pneumophila* in VBNC state has been recovered from water sources after the disinfection process, by co-culturing with *Acanthamoeba polyphaga* (85). Since freshwater amebae are the natural hosts of *L. pneumophila*, the trophozoite forms play a vital role in its ecology and infectivity to humans. It has recently been shown that chlorination induces cell permeabilization, size reduction, and likely concentration of intracellular thiol in acanthamoebae population (86), which in turn may favor an increase in the number of *L. pneumophila* within the host.

Using the fluorescent antibody technique, VBNC forms of *V. cholerae* was detected in zooplankton samples collected from the estuaries of rivers Anil and Bacanga in São Luis, Maranhão, Brazil (87). During cholera outbreak periods, *V. cholerae* O1 in VBNC state was detected in samples collected from rivers in Tucumán, Argentina by direct immunofluorescence. However, no correlation between its presence and physico-chemical parameters could be seen (88). In Bangladesh, animal models and microcosm studies with *V. cholerae* O1 did not support the hypothesis that VBNC state may have a role to play in the transmission of cholera (89).

Viability of *H. pylori* was found to be extended over 24 h in chlorinated drinking water (~1 mg/l of free chlorine) and the organism changed its morphology mostly into coccoid forms. Expression of *vacA* gene and levels of 16S rRNA were constant at all test time points (66). These results demonstrate that *H. pylori* continue to survive in the VBNC state in chlorinated drinking water and hence can cause infection upon its consumption. Enterotoxigenic *E. coli* can also enter into a VBNC state during stressful conditions and continue to remain infectious after long-term incubation in water (12). *E. faecalis* has been used as one of the indicators of fecal pollution in recreational water. The VBNC status of this organism severely affects its proper enumeration by culture-based microbial water quality assessment methods (90).

## Resuscitation-Promoting Factor

During the process of infection, many pathogens struggle to survive in the infection sites, especially against the onslaught of many substances such as molecules produced and secreted by the host, other competing microbes and exogenous antibacterial drugs that may be given during the treatment. These factors may influence the pathogens to enter into the VBNC state, making them metabolically inactive. However, they can be resuscitated under particular conditions where they can express their virulence attributes. Recently, it has been discovered that in resuscitating VBNC cells a bacterial cytokine, known as resuscitation-promoting factor (Rpf), which was first described as a secretory protein of *Micrococcus luteus* (91) and may have either autocrine (by the same cells that produce them) or paracrine (may act on the nearby cells) signaling functions plays an important role.

Resuscitation-promoting factor or its homologs have also been reported in other bacterial species including *Mycobacterium* spp., *Corynebacterium* spp., *Streptomyces* spp., *L. monocytogenes, Tomitella biformata*, and *S. enterica* serovar Typhimurium (1, 92–94). Rpf-mediated resuscitation of *M. tuberculosis* has been demonstrated in several studies (95, 96). *M. tuberculosis* comprises five Rpf-like proteins, of these, RpfA-E have been shown to be involved in the resuscitation of this pathogen from the VBNC state through a process linking, hydrolysis of the peptidoglycan by Rpfs and partnering proteins (97).

Resuscitation-promoting factor with predicted peptidoglycan hydrolytic activities was found to contribute to the revival of VBNC cells (98). In bacteria, the Rpf domains may have a different structure and activity, but all the Rpfs include a functional conserved domain with lysozyme-like activity. The mechanism involving Rpf in the resuscitation of VBNC cells probably involves its ability to cleave the cell wall components, thereby producing peptidoglycans that can function as signaling molecules for the growth initiation (99). This suggests that the stimulation of dormant cells requires peptidoglycan hydrolysis, which either alters the mechanical properties of the cell wall to enable cell division or discharges lysis products that function as anti-dormancy signals (100). *E. faecalis* in the VBNC state also show highest levels of peptidoglycan O-acetylation and specific autolysis (101). A protein termed Rpf interacting protein A (RipA) enables these proteins to synergistically degrade peptidoglycan to facilitate growth. Furthermore, it is thought that the simultaneous action of Rpfs with RipA and other peptidoglycan hydrolases might produce muropeptides that could exert diverse biological effects through host and/or bacterial signaling pathways, which in the latter case involves serine/threonine protein kinases (99).

Resuscitation-promoting factors of Gram-positive bacteria function as peptidoglycan hydrolases (91) that are likely to be involved in cell-wall digestion and cell division (102). The Rpfs of Gram-negative bacteria belong to a distinctly different class of proteins (named YeaZ) that have unknown biochemical functions (92, 103). The YeaZ homologs expressed by *E. coli* and *S*. Typhimurium have been shown to be essential for their survival (103, 104).

It is well known that all the Rpfs share a conserved domain of about 70 amino acids and possess a lysozyme-like activity. Structural studies of this conserved domain suggest that the Rpfs could be considered a c-type lysozyme and lytic transglycosylase. Recently, a novel class of nitrophenylthiocyanate (NPT) inhibitors of the muralytic activity of Rpf has been reported, which opens a new approach to the study of cell-wall hydrolyzing enzymes. It has also been observed that peptidoglycan-dependent resuscitation activity can be suppressed by means of an inhibitor (4-benzoyl-2-nitrophenylthiocyanate) specific for Rpf (98). This compound is now viewed as a promising scaffold for the generation of therapeutic agents targeting latent tuberculosis.

## Conclusion

The resuscitation of VBNC state is not only important in the field of medicine, but also for several industrial processes to improve biodegradation and flocculation (105). Although a considerable amount of work is going on in this area, specific mechanism responsible for the transition of bacteria to the VBNC state or its revival to culturability remain obscure. There is an urgent need for further research involving a combination of cultural and molecular approaches to understand the phenomenon, as the formation of VBNC state in bacteria and its resuscitation pose a great threat to public health. For the same reason, further improvement of the detection methods involving fluorescent reporter dyes, microfluidic, microelectromechanical systems, and time-lapse fluorescence microscopy remains an urgent necessity.

## Conflict of Interest Statement

The authors declare that the research was conducted in the absence of any commercial or financial relationships that could be construed as a potential conflict of interest.
